# Biological Evaluation and Molecular Docking of Protocatechuic Acid from *Hibiscus sabdariffa* L. as a Potent Urease Inhibitor by an ESI-MS Based Method

**DOI:** 10.3390/molecules22101696

**Published:** 2017-10-11

**Authors:** Sherif T. S. Hassan, Emil Švajdlenka

**Affiliations:** 1 Department of Natural Drugs, Faculty of Pharmacy, University of Veterinary and Pharmaceutical Sciences Brno, Palackého tř. 1946/1, 612 42 Brno, Czech Republic; svajdlenkae@vfu.cz; 2 Department of Applied Ecology, Faculty of Environmental Sciences, Czech University of Life Sciences Prague, Kamýcká 129, 165 21 Praha 6-Suchdol, Czech Republic

**Keywords:** ESI-Mass spectrometry, urease inhibitors, molecular docking, *Hibiscus sabdariffa* L., protocatechuic acid, cytotoxicity

## Abstract

Studies on enzyme inhibition remain a crucial area in drug discovery since these studies have led to the discoveries of new lead compounds useful in the treatment of several diseases. In this study, protocatechuic acid (PCA), an active compound from *Hibiscus sabdariffa* L. has been evaluated for its inhibitory properties against jack bean urease (JBU) as well as its possible toxic effect on human gastric epithelial cells (GES-1). Anti-urease activity was evaluated by an Electrospray Ionization-Mass Spectrometry (ESI-MS) based method, while cytotoxicity was assayed by the MTT method. PCA exerted notable anti-JBU activity compared with that of acetohydroxamic acid (AHA), with IC_50_ values of 1.7 and 3.2 µM, respectively. PCA did not show any significant cytotoxic effect on (GES-1) cells at concentrations ranging from 1.12 to 3.12 µM. Molecular docking study revealed high spontaneous binding ability of PCA to the active site of urease. Additionally, the anti-urease activity was found to be related to the presence of hydroxyl moieties of PCA. This study presents PCA as a natural urease inhibitor, which could be used safely in the treatment of diseases caused by urease-producing bacteria.

## 1. Introduction

Over the past few decades, treatment of bacterial infections caused by urease-producing bacteria has gained much interest. This is due to the extensive use of antibiotics, which has created antibiotic resistance, associated with other adverse effects that have led to the failure of current treatment strategies [1,2]. Therefore, it very crucial to find alternative strategies to overcome such problems. Urease (EC 3.5.1.5, urea amidohydrolase) is a nickel-dependent metalloenzyme that catalyzes the hydrolysis of urea to ammonia and CO_2_ or carbamate [3,4]. A variety of ureases are found in nature, including higher plants, bacteria, fungi, and soil enzymes. Urease has been reported to be a prominent virulence determinant in the pathogenesis of many clinical conditions which are detrimental for human and animals, as well as for agriculture [5,6]. This enzyme is known to be one of the major causes of diseases induced by *Helicobacter pylori* (*H. pylori*), in which it allows bacteria to colonize and survive at low pH in the stomach. Urease has been shown to contribute to the pathogenesis of urolithiasis, pyelonephritis and hepatic encephalopathy, hepatic coma, and urinary catheter encrustation [7,8]. Currently, one of the most important alternative strategies for the treatment of urease-related diseases is the use of urease inhibitors [9,10]. Numerous substances either from natural, synthetic, or semisynthetic sources have been reported to exert anti-urease activity, including, but not limited to, hydroxamic acids, phosphoramidates, urea derivatives, quinones, phenolic compounds, terpenoids, and alkaloids [11,12,13,14,15]. In clinical practice, acetohydroxamic acid (AHA) was the only drug that has been approved by the U.S. Food and Drug Administration for the treatment of infections caused by urease-producing bacteria. However, some limitations associated with severe side effects, such as teratogenicity, psycho-neurological, and musculo-integumentary symptoms, have resulted in limited use of this inhibitor [16,17]. Bioactive components of natural origin have been under enormous investigation as potential effective urease inhibitors with good bioavailability, less toxicity, better stability, and less undesirable side effects [17].

*Hibiscus sabdariffa* L. (*H. sabdariffa*, Roselle) is an annual dicotyledonous, herbaceous shrub belonging to the family Malvaceae. This plant is widely distributed in tropical and subtropical regions of Africa, Asia, and South America [18]. *H. sabdariffa* has been shown to exert various biological properties including antimicrobial properties [19,20]. *H. sabdariffa* aqueous extracts are reported to possess direct inhibitory properties against *H. pylori* [21] as well as anti-enzymatic properties against urease [22].

Protocatechuic acid, a catecholtype *O*-diphenol phenolic acid (PCA; 3,4-dihydroxybenzoic acid), an important bioactive compound of *H. sabdariffa*, has elicited the interest of researchers in the last few years because of the variety of its biological activities related to human health [23,24,25]. This study has been designed to evaluate the anti-jack bean urease (JBU) activity of PCA and to evaluate its safety as an effective urease inhibitor. Molecular docking simulation studies were employed to understand the interaction of PCA with the urease active site.

## 2. Results

### 2.1. Identification and Determination of Concentration of PCA in H. sabdariffa

**Figure 1:**
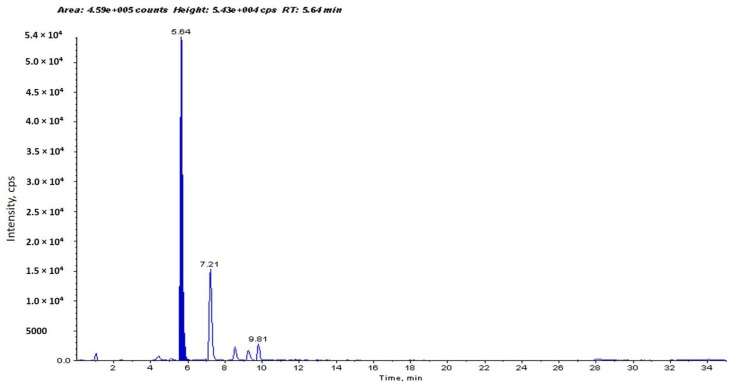
High-performance liquid chromatography-mass spectrometry (HPLC-MS) chromatogram of an aqueous extract of *H. sabdariffa* shows identification (two MRM transitions *m*/*z* 153→109 and *m*/*z* 153→91, at retention time 5.64 min) and determination of the concentration of protocatechuic acid (PCA). The other peaks are probably its isomers resulting from multiple reaction monitoring (MRM) transitions (at different retention times). PCA was determined to be 94.1 µg/g dry weight of *Hibiscus* calyces (0.0941%).

**Figure 2:**
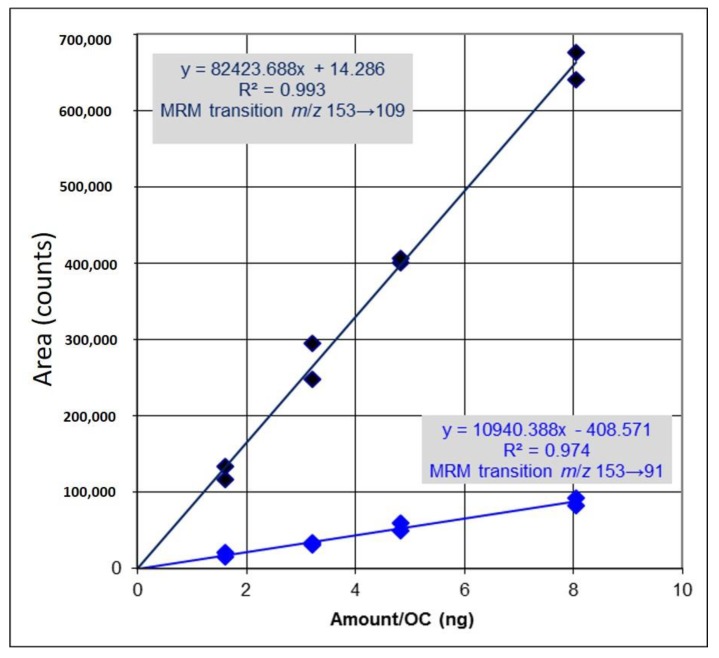
Calibration curve showing the quantification of protocatechuic acid (PCA) in *Hibiscus sabdariffa* water extract. An external calibration method with standard PCA (two MRM transitions *m*/*z* 153→109 and *m*/*z* 153→91) was used for quantification. OC: Amount (nanogram) in-column.

The concentration of PCA in *Hibiscus* calyces water extract was determined to be 94.1 µg/g dry weight of *Hibiscus* calyces (0.0941%) (Figure 1). PCA was quantified using an external calibration method with standard PCA (Figure 2).

### 2.2. Urease Inhibition Studies and Determination of IC_50_

**Figure 3:**
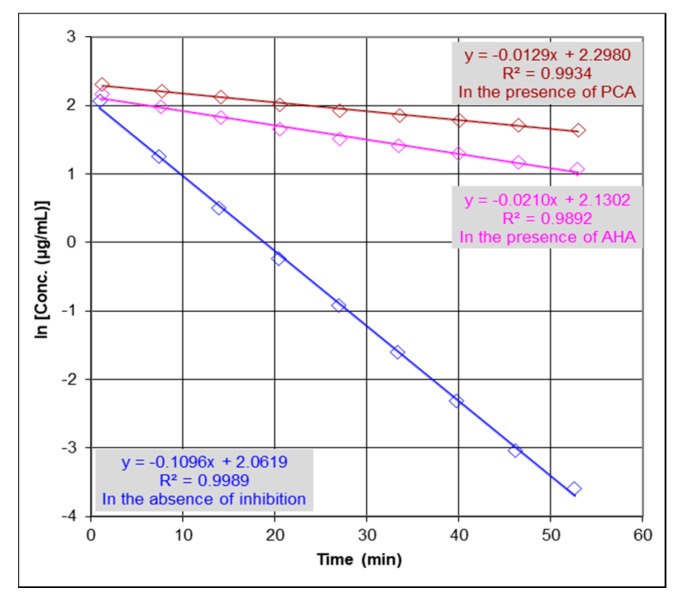
Anti-urease activity of protocatechuic acid (PCA) and acetohydroxamic acid (AHA) evaluated by an Electrospray Ionization-Mass Spectrometry (ESI-MS) based method. k_0_ is the reaction rate constant in the absence of inhibition [●] (k_0_ = 0.1096/min) and k is the reaction rate constant in the presence of PCA [●] and AHA [●] (k = 0.0129 and 0.0210/min, respectively). Concentrations changes of urea are presented as logarithms of concentration. IC_50_ values for PCA and AHA were determined to be 1.7 and 3.2 µM, respectively. The precision of the time course analysis was calculated as relative standard deviation (RSD) (%) of multiple measured slopes (lower than 10%). For clarity of figure, multiple measurements have not been presented.

The anti-urease activity of PCA was evaluated by an Electrospray Ionization-Mass Spectrometry (ESI-MS). Anti-urease activity was determined based on the detection of urea depletion in the absence and presence of inhibitors by monitoring the catalytic reaction through detection of the concentration changes of urea. Figure 3 shows that the reaction rate constant in the presence of inhibition (k) is lower than the reaction rate constant in the absence of inhibition (k_0_), where k in the presence of PCA (k = 0.0129/min) and in the presence of acetohydroxamic acid (AHA; used as standard urease inhibitor) (k = 0.0210/min) and (k_0_ = 0.1096/min) at concentrations of 253.0 µM for urea and 33.8 µg/mL for urease, and concentrations of inhibitors (PCA = 13.0 μM and AHA = 13.3 μM). The percent inhibition is defined as the percent reduction of the reaction rate constant (k) as compared with the rate constant in the absence of an inhibitor (k_0_). Thus, % activity is determined as $\frac{k}{k_{0}}$ and % Inhibition is 1 − % activity. Therefore, to determine IC_50_ values for PCA and AHA, the following equation % activity = $\frac{k}{k_{0}}=1-\frac{\left[I\right]}{\left[I\right]\;+{\mathrm{IC}}_{50}}=\frac{{\mathrm{IC}}_{50}}{\left[I\right]\;+{\mathrm{IC}}_{50}}\;$, where [I] is the concentration of the inhibitor, was used. The results showed that PCA exerted notable inhibition properties against JBU compared with that of AHA, with IC_50_ values of 1.7 and 3.2 µM, respectively. The determined IC_50_ value for AHA compared favorably with previous data reported in literature (IC_50_ values ranging from 3.0 to 5.0 µM) [12,22,26].

### 2.3. Effect of PCA on GES-1 Viability

**Figure 4:**
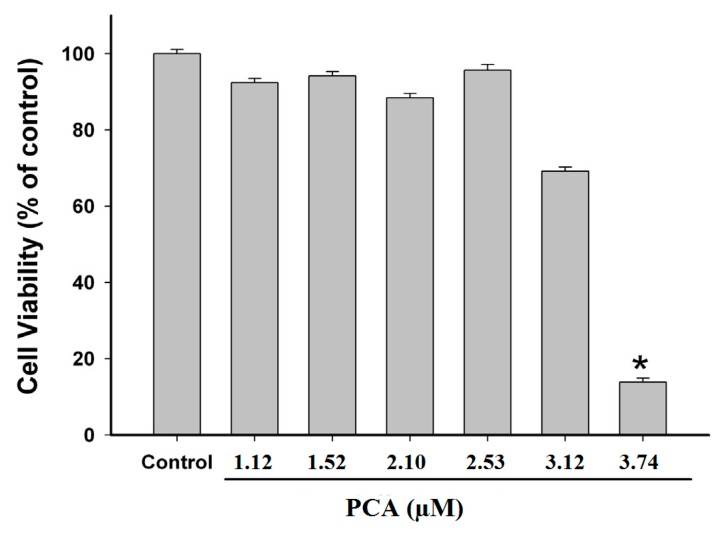
Effect of protocatechuic acid (PCA) on human gastric epithelial (GES-1) cell viability at different concentrations ranging from 1.12 to 3.74 µM. Values given are means ± S.E.M. of three independent measurements. * *p* < 0.05, indicates significant different compared with the control group.

To evaluate the possible cytotoxic effect of PCA, various concentrations of PCA were administered in the cytotoxicity assay using human gastric epithelial cells (GES-1). The results demonstrated that PCA at concentrations of 1.12, 1.52, 2.10, 2.53 and 3.12 µM did not exhibit significant cytotoxicity to GES-1 cells, when compared with control group (Figure 4). Based on our results, we may suggest that PCA could be used safely as a natural urease inhibitor. However, further investigations should be carried out to confirm its safety in vivo.

### 2.4. Molecular Docking Studies

**Figure 5:**
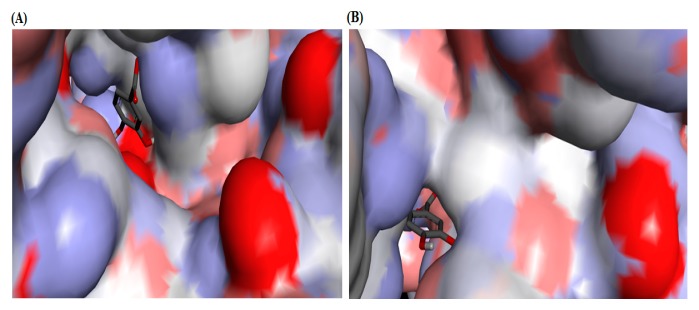
Surface representation of the active-site flap of urease with protocatechuic acid (PCA), shown at the entrance of the binding pocket. (**A**) Jack bean urease (JBU; PDB code: 3LA4); (**B**) *Helicobacter pylori* urease (HPU; PDB code: 1E9Y).

To understand the interaction of PCA with the urease active site, PCA was subjected to molecular docking simulation studies that were performed by using PyRx docking tool through Autodock VINA software. Molecular docking of PCA into the binding site of jack bean urease (JBU) and *Helicobacter pylori* urease (HPU) was performed based on the published structures of JBU (PDB code: 3LA4) and HPU (PDB code: 1E9Y) and complex structures, and the best possible binding modes of PCA were depicted as an enzyme surface model (Figure 5). The docking scores of PCA with JBU and HPU are given in Table 1, where the generated docked complexes were examined based on binding affinity values (kcal/mol) and bonding interaction patterns (hydrogen, hydrophobic, and electrostatic).

**Figure 6:**
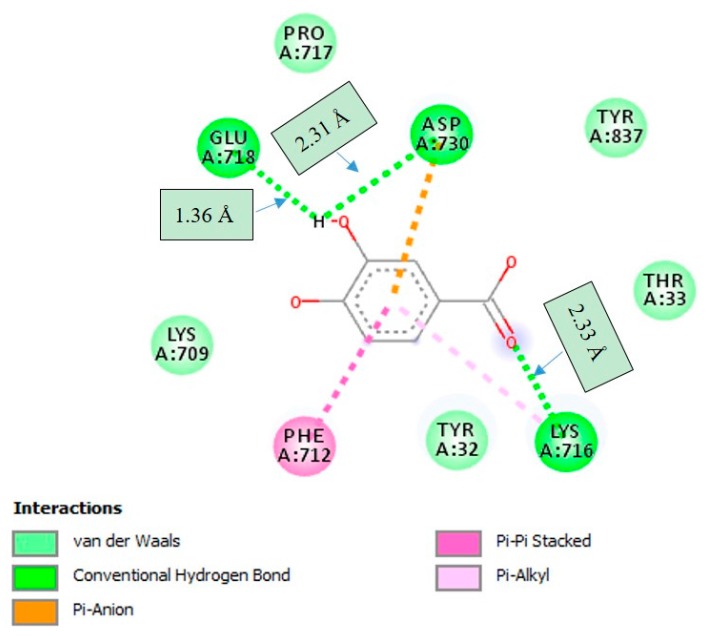
2D interaction diagram of protocatechuic acid (PCA) in the active site of jack bean urease (JBU; PDB code: 3LA4). Only those amino acid residues involved in JBU stabilization are shown. Hydrogen bonding interactions with amino acid residues at corresponding distances are shown.

PCA was found to make strong hydrogen bonding interactions with amino acid residues GLU-718, ASP-730, and LYS-716 with distances of 1.36 Å, 2.31 Å and 2.33 Å, respectively, which were located on the active-site mobile flap of JBU. A number of important interactions were observed (Figure 6). PCA was not found to interact with the active-site Ni^2+^. Interestingly, it was found that hydroxyl groups of PCA affected the molecular conformation interacting with JBU, which enhanced the binding with GLU-718 and ASP-730 amino acid residues through the hydrogen bonding interactions, and hence resulted in lowered enzymatic activity.

**Figure 7:**
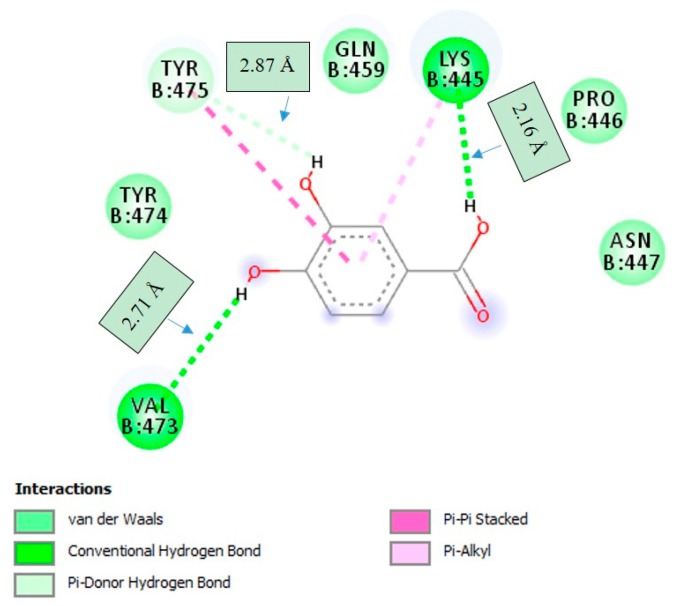
2D interaction diagram of protocatechuic acid (PCA) in the active site of *Helicobacter pylori* urease (HPU; PDB code: 1E9Y). Only those amino acid residues involved in HPU stabilization are shown. Hydrogen bonding interactions with amino acid residues at corresponding distances are shown.

The docking studies also showed that the best orientation of PCA in the active pocket of HPU was formed by hydrogen bonding with the amino acid residues of LYS-445 and VAL-473 with distances of 2.16 Å and 2.71 Å, respectively, while with TYR-475 formed Pi-donor hydrogen bonding with distance of 2.87 Å (Figure 7). As shown in Figure 7, several important interactions were also observed. Hydroxyl groups of PCA were found to be an important factor that affect the molecular conformation interacting with HPU, which enhanced the binding with LYS-445 and VAL-473 through the hydrogen bonding interactions. The docking results did not confirm any interactions of PCA with the active-site Ni^2+^.

## 3. Discussion

In recent years, inhibition of urease has played an important role in medicinal practices and in agriculture as well. High concentrations of ammonia arising from urease-catalyzed reaction and accompanying pH elevation have important negative effects on humans and agriculture [27,28]. Thus, inhibition of urease may open new strategies for the treatment of diseases associated with urease-producing bacteria [27]. Recently, our research group has developed a method that utilizes mass spectrometry to reproducibly measure urease activity and the inhibition properties of natural products and synthetic and semisynthetic drugs by an ESI-MS based assay [22]. The results reported that *H. sabdariffa* water extract showed remarkable anti-urease activity, which was believed to be more likely to correlate with its rich content of phenolic acids and polyphenols. In this study, as part of our ongoing search of natural sources for therapeutic and preventive agents for urease inhibition, the inhibitory effect of PCA from *H. sabdariffa* on JBU was investigated. PCA showed notable inhibitory properties against urease compared with that of AHA as a reference urease inhibitor.

Several in vitro studies have reported that PCA is non-toxic to various human cells at concentrations lower than 2.0 mM [24,29,30]. However, it is very crucial to take into account that the concentration of PCA in vivo does not proportionally correspond to the quantity consumed, but is higher, because PCA is the main metabolite of several complex polyphenolic compounds [31]. It has been reported that many anthocyanins and procyanidins after oral administration are metabolized to PCA by intestinal microflora and can be detected in human blood and urine at a concentration higher than the parent anthocyanins or procyanidins [32,33]. In addition, due to the low absorbtion by oral route, PCA is non-toxic and hence considered as a relatively safe compound for oral administration [25]. Our study revealed that PCA did not exert a significant cytotoxic effect on GES-1 cells, and thus could be considered as a safe urease inhibitor.

Molecular docking plays an essential role in the rational design of drugs, being a useful tool which reasonably predicts the best orientation of one molecule within the putative target. It is known that the macromolecular structure of JBU is different to that of HPU, although the active site structure of JBU is similar to that of HPU containing a bi-nickel center [34]. Based on these considerations, the structural distinction between JBU and HPU was believed to be closely associated with the urease inhibition difference observed for PCA, such as the differences of amino acid residues in the active site architectures of both JBU and HPU, and the distances. It has been reported that amino acid residues such as GLU, ASP, LYS, VAL, and TYR were found in the active site architectures of JBU and HPU, which form hydrogen bonding interactions with the ligand, are responsible for the inhibition of enzyme activity [35,36]. In our study, the protein–ligand interaction profile revealed the importance of the hydrogen bonding between the hydroxyl groups of PCA and the amino acid residues of the active sites of JBU and HPU. Additionally, the docking results showed that PCA has better binding affinity than AHA in both JBU and HPU. Many medicinal chemicals can exchange protons with their environment, resulting in various ionization and tautomeric states, collectively known as protonation states. Considering the importance of protonation state and the overal charge in molecular docking studies, factors such as pH and the local molecular environment of the compound which affect the protein–ligand interactions should be taken into account. Although there are often many potential protonation states for a medicinal chemical, usually only a small number are present at significant levels under physiological conditions [37,38].

Based on the above-mentioned findings, results obtained from molecular docking analyses are in good correlation with in vitro results obtained by this study, which confirm the inhibitory properties of PCA against JBU.

## 4. Materials and Methods

### 4.1. Identification and Determination of Concentration of PCA in H. sabdariffa

Identification and determination of concentration of PCA in *Hibiscus* calyces water extract were assayed using HPLC-MS analyses as previously described [22].

### 4.2. Enzyme, Substrate, Inhibitors, and Reagents

Urease (Jack bean urease; JBU-type III from *Canavalia ensiformis*), urea, acetohydroxamic acid (AHA; standard urease inhibitor), and PCA were purchased from (Sigma Aldrich, Prague, Czech Republic). All chemical reagents were obtained from commercial suppliers and used without further purification.

### 4.3. ESI-MS

Anti-urease activity was evaluated using a system pump-injector (Agilent 1200, Berlin, Germany) coupled with a Sciex-3200QTRAP–hybrid triple quadrupole/linear ion trap mass spectrometer (Toronto, ON, Canada) fitted with Electrospray Ionization (ESI). The system runs in flow injection analysis (FIA) mode without a HPLC column. The operational parameter settings were as follows: curtain gas (CUR), 25 psi; nebulizer gas (GS1), 50; auxiliary gas (GS2), 40; declustering potential (DP), 15 V; ion spray voltage, −4000 V; turbo temperature (TEM), 450 °C. MS in positive ion mode was used in multiple reaction monitoring (MRM) analysis for detection and quantitation of urea with transition *m*/*z* 61→44. 0.1% HCOOH and 1 mM HCOONH_4_ were used as mobile phases with the flow rate set at 0.5 mL/min. The injection volume was 10 μL.

### 4.4. Anti-Urease Activity by ESI-MS-Based Assay

Urease inhibitory activities of PCA and AHA were evaluated by an Electrospray Ionization-Mass Spectrometry (ESI-MS) based assay based on the detection of urea depletion in the absence and presence of inhibitors by monitoring catalytic reaction through detection of the concentration changes of urea as previously described [22]. Briefly, enzymatic reaction was performed by pre-incubating JBU in 1 mM HCOONH_4_ buffer (pH = 7.5) with PCA at concentration of 13.0 μM and AHA at concentration of 13.3 μM for 105 min to reach binding equilibrium, followed by adding urea (as a substrate) at concentration of 253 µM. The solutions were directly injected automatically into FIA system and the concentration changes of urea were monitored. For the analysis of the kinetics of substrate depletion by ESI-MS, areas (total counts) under peaks for substrate in the FIA record were integrated.

### 4.5. Cell Viability Assay

To evaluate the cytotoxicity of PCA on human gastric epithelial cells (GES-1; were kindly obtained from the Motol University Hospital, Prague, Czech Republic), cell viability was measured by MTT assay as previously described [39]. Briefly, the cells were treated with PCA at different concentrations (1.12, 1.52, 2.10, 2.53, 3.12 and 3.74 µM). After drug treatment, 20 μL of MTT solution (final concentration, 1.0 mg/mL) was added into each well, and the cells were incubated at 37 °C for 4 h. The culture medium was removed, and the formazan crystals were dissolved with 150 μL of Dimethyl sulfoxide (DMSO). The optical density of each well was measured using a microplate reader (FLUOstar OPTIMA, BMG Labtech, Berlin, Germany) at 570 nm. Cell viability was expressed as percentage of nontreated control.

### 4.6. Molecular Docking Analyses

To evaluate the possible interaction mode between PCA and plant and bacterial ureases, the molecular docking experiments were performed using PyRx docking tool through Autodock VINA software [40]. The three-dimensional (3D) crystal structure of jack bean urease (JBU; PDB code: 3LA4) and *Helicobacter pylori* urease (HPU; PDB code: 1E9Y) were obtained from the RCSB Protein Data Bank. JBU and HPU had a resolution of 2.05 Å and 3.00 Å, respectively. The standard three-dimensional (3D) structure (PDB format) of PCA was obtained from chem3D Ultra 8.0 software (Chem3D Ultra 8.0, CambridgeSoft Corporation, Cambridge, MA, USA) and was prepared for docking by minimizing its energy. The macromolecules of JBU and HPU were uploaded in the PyRex tool which automatically removes the solvent molecules followed by hydrogen addition and gasteiger charges calculations. The SDF file of the PCA was uploaded in PyRex tool linked with Autodock VINA. The receptor and SDF file of PCA were converted into pdbqt format. The Grid center was positioned on the active sites of the both JBU and HPU. The default exhaustiveness value was used to maximize the binding conformational analysis. The generated docked complexes were examined based on binding affinity values (kcal/mol) and bonding interaction patterns (hydrogen, hydrophobic, and electrostatic). To establish the protonation state of the urease-PCA complex and the overall charge, docking calculations were performed using a previously established protocol [41]. The graphical depictions of all the docked complexes were accomplished by Discovery studio visualizer version 4.0 (BIOVIA, San Diego, CA, USA) [42] and PyMOL version 1.7.2 [43] software (DeLano Scientific LLC, San Carlos, CA, USA).

## 5. Conclusions

For decades, medicinal plants and their biologically active compounds have played a crucial role in traditional folk medicine as a first line treatment strategy of various diseases. In this study, PCA has been identified as a prominent urease inhibitory constituent from *H. sabdariffa*. In addition, PCA did not show any significant cytotoxic effect on (GES-1) cells at concentrations ranging from 1.12 to 3.12 µM. This indicates that PCA could be used safely as a natural urease inhibitor. It can be inferred from the results of in vitro and molecular docking studies that PCA has good potential for further development as a promising therapeutic approach for the treatment of urease-related diseases. Also, it should be considered in further studies, especially in vivo, that the use of PCA as urease inhibitor in combination therapy with antibiotics and proton pump inhibitors is another potential option for the treatment of diseases caused by urease-producing bacteria.

## Figures and Tables

**Table 1:** Docking results of Jack bean urease (JBU; PDB code: 3LA4) and *Helicobacter pylori* urease (HPU; PDB code: 1E9Y) with protocatechuic acid (PCA) and acetohydroxamic acid (AHA; standard urease inhibitor).

Compound	JBU Binding Affinity (kcal/mol)	HPU Binding Affinity (kcal/mol)
PCA	−5.8	−5.9
AHA	−4.8	−4.9

Docking studies were performed using PyRx docking tool through Autodock VINA software.
